# Influence of different industrial resource profiles on taxonomical richness and community structure of insects populations

**DOI:** 10.1038/s41598-025-32865-3

**Published:** 2026-01-06

**Authors:** Mohamed A. M. Shahat, Mohamed A. M. El-Tabakh, Yasser I. Hamza, Ahmed M. A. Elnaggar, Wesam M. A. Ward, Heba F. Abd-Elkhalek, Ahmed Z. I. Shehata

**Affiliations:** 1 Zoology Department, Faculty of Science, Al-Azhar University, Cairo, Egypt; 2 Entomology Department, Faculty of Science, Benha University, Benha, Egypt

**Keywords:** Biodiversity indices, Food industries, Environmental changes, Insects, Ecology, Ecology, Zoology

## Abstract

Industrial activity has significant effects on terrestrial biota and insect community which are considered a significant biological indicator of alterations in the environment. This research examined insect diversity, dominance, and abundance over two years on eight factory types (both non-food and food-processing) in 6th of October City, Egypt. To describe insect community structures and determine the ecological drivers of observed patterns, the study used a variety of biodiversity indices, such as Taxa_S, Shannon_H, Simpson_1-D, Menhinick, Margalef, Fisher’s Alpha, Evenness, Equitability_J, Dominance_D, Brillouin, Berger-Parker, and ACE. Due to the abundance of organic waste, the results consistently demonstrated that food-processing factories, especially the ice cream and cheese factories, supported the highest taxonomic richness and diversity. Conversely, the car’s factory exhibited the lowest diversity, though it demonstrated unexpected improvements in evenness in 2022, possibly indicating microhabitat development or reduced stressors. Despite maintaining low variety, the chips & corn factory consistently recorded the highest insect abundance, indicating substantial dominance by a small number of highly adapted species. The cycle of seasons and thus the massed population in the onion factory was unconventional due to the unpleasant nature of onion byproducts. The ice cream factory carried a more balanced community as compared to the chocolate and Meat Factories that had high Berger-Parker indices. In most places, spring was a season of seasonal highs in diversity and abundance. The influence of personal products of industry turns out with the rich and unreasonable preponderance of Coleoptera (Ptinidae, Nitidulidae) and over-abundance of Diptera, in particular, Muscidae and Drosophilidae. Such findings evidence the role of industry in greatly influencing insect ecology and show how a tailored seasonally adjustable whole integrated pest management protocol dependent upon the resource availability, ecological considerations specific to a particular factory are needed.

**Supplementary Information:**

The online version contains supplementary material available at 10.1038/s41598-025-32865-3.

## Introduction

Industry is the foundation of the global economy and a major contribution to any country’s progress. Despite its importance, its detrimental impact has been amplified by a lack of accurate estimates of the impact on the adjacent terrestrial biota. Industrial pollution is one of the most harmful anthropogenic impacts on any environment because of its long-term consequences and growing weight with time^1–3^.

Biodiversity serves as a crucial ecological indicator for evaluating both global and local environmental changes, as well as the sustainability of developmental activities^4^. It describes the diversity of life on all scales and the mechanisms that control them to represent the interactions between genes, species, and ecosystems^5–7^. It is synonymous with species diversity and is measured by the number of species present in a given area, referred to as species richness^8,9^.

Insects serve as a valuable ecological tool for evaluating environmental changes within ecosystems due to their high diversity and significant influence on terrestrial ecosystems compared to other organisms^10–12^.

So, the aim of this study was to investigate how different industrial environments and activities influence insect population dynamics, diversity, and community structure across different factory types over four seasons at 6th October City, Egypt, over a two-year period. By comparing food-processing industries (such as ice cream and cheese factories) with non-food industrial settings (such as car factories).

## Materials and methods

### Study area

6th October city is one of the new cities, situated in the South-west of the Greater Cairo desert margin, about 32 km from Cairo governorate, at the intersection of a longitude (30º, 45ʹ) in the east and latitude (30º, 00ʹ) to the north. It is also located within Giza administrative borders and has a unique geographical location near to the greater Cairo region^13,14^.

The study was conducted over two full years, 2021 and 2022, in the Sixth of October Industrial Zone in 6th of October City, Giza Governorate, Egypt. Insect samples were collected from seven factories representing different food industries and compared with a non-food factory to determine if insects are more attracted to specific industries than others.

The factories activities included: Ice cream factory, Cheese factory, Biscuit factory, Chocolate factory, Meat factory, Chips and snacks factory, Onion factory, and cars factory.

In this study, not only were the different industries considered, but also the different locations of these factories within the collection area. The ice cream factory was in the First Industrial Zone, while the cheese and biscuit factories were in the Second Industrial Zone. The chocolate factory was in the Third Industrial Zone, the meat factory in the Fourth, the chips factory in the Sixth, the onion factory in the Industrial Zone Extension, and the automotive factory was also in the Fourth Industrial Zone.

### Sample collection

Insect samples were collected from light traps inside the production halls and from within the warehouses and storage areas of the eight factories. Samples were also gathered from ground glue traps intended for rodents, which were found to contain a considerable number of insects, including both crawling and flying species.

Samples were collected over two full years, representing all four seasons (spring, summer, autumn, and winter). This involved two visits per month for each season, totaling 24 visits per year and 48 visits over the two-year period.

Each time samples were collected, they were preserved in 70% alcohol for transport to the Animal House laboratories at the Faculty of Science, Al-Azhar University, Nasr City, Cairo. The exception was butterflies, which were preserved dry to protect their wing scales, as these are crucial for classification.

### Statistical analysis

The statistical software SPSS V.22 was used to code and input the data. Data were tested for satisfying assumptions of parametric tests; continuous variables were subjected to Shapiro-Wilk and Kolmogorov-Smirnov tests for normality. Probability and percentile data were standardized for normality using Arcsine Square Root. Data were presented as mean and standard deviation. ANOVA analyses were done for the investigated sites regarding the recorded insects abundance; analysis was evaluated using at least three replicates of traps at each site; post-hoc analysis was assessed using Tukey pairwise comparison using MiniTab V 14; P-values were considered significant at < 0.05.Data were visualized when possible, using R studio V 2022.02.4.

The following biodiversity indices were used to assess species diversity and community structure across the study sites. Each index was calculated using standard formulas, as outlined below.

1. Taxa_S (Species Richness).

Species richness, denoted as Taxa_S, was used to quantify the total number of species observed in the community. This is calculated as:

$${\text{Taxa}}\_{\text{S}}\,=\,{\text{Total number of species present}}$$

2. Simpson’s index (1-D)

Simpson’s Index (1-D) was used to measure community diversity, considering both species richness and evenness. The formula for Simpson’s Index is:

$${\text{D}}\,=\,{\text{1 }} - {\text{ }}\sum {\text{ }}\left( {{\text{n}}\_{\text{i }}\left( {{\text{n}}\_{\text{i }} - \,{\text{1}}} \right){\text{ }}/{\text{ N }}\left( {{\text{N }} - \,{\text{1}}} \right)} \right)$$

where n_i is the number of individuals of species i, and N is the total number of individuals in the community.

3. Shannon index (H)

The Shannon Index was employed to quantify the entropy or uncertainty in the community, accounting for both species richness and evenness. The formula is:

$${\text{H}}'{\text{ }}={\text{ }} - \sum {\text{ }}\left( {{\text{p}}\_{\text{i }}*{\text{ ln p}}\_{\text{i}}} \right)$$

where p_i is the proportion of individuals in species i.

4. Menhinick index

The Menhinick Index measures species richness relative to the square root of the total number of individuals. It is calculated as:

$${\text{M}}\,=\,{\text{S }}/{\text{ }}\surd {\text{N}}$$

where S is the number of species, and N is the total number of individuals.

5. Margalef index

The Margalef index quantifies species richness adjusted for sample size. It is given by

$${\text{D }}={\text{ }}\left( {{\text{S }} - \,{\text{1}}} \right){\text{ }}/{\text{ ln N}}$$

where S is the number of species, and N is the total number of individuals.

6. Individuals (N)

The total number of individuals in the community, N, was counted as the sum of individuals across all species:

$${\text{N }}={\text{ }}\sum {\text{ n}}\_{\text{i}}$$

where n_i represents the number of individuals of species i.

7. Fisher’s Alpha

Fisher’s Alpha is an index used to estimate the number of species based on the number of rare species. The formula is:

$${\text{a}}={\text{ }}\left( {{\text{S }} - \,{\text{1}}} \right){\text{ }}/{\text{ }}\left( {{\text{ln }}\left( {\text{N}} \right){\text{ }} - {\text{ ln }}\left( {{\text{N }} - {\text{ S}}} \right)} \right)$$

where S is the number of species, and N is the total number of individuals.

8. Evenness (e^H/S)

The Evenness Index, based on the Shannon Index, measures how evenly individuals are distributed across species. The formula is:

$$E={e^ \wedge }{H^\prime }/S$$

where H’ is the Shannon Index, and S is the number of species.

9. Equitability (J)

Equitability measures the evenness of species abundance in the community and is calculated as:

$${\text{J}}{\mkern 1mu} = {\mkern 1mu} {{{\text{H}}^{\prime } } \mathord{\left/ {\vphantom {{{\text{H}}^{\prime } } {{\text{ln S}}}}} \right. \kern-\nulldelimiterspace} {{\text{ln S}}}}$$

where H’ is the Shannon Index, and S is the number of species.

10. Dominance (D)

Dominance quantifies the extent to which a few species dominate the community. It is calculated using the formula:

$$D=\sum {p\_} {i^ \wedge }2$$

where p_i is the proportion of individuals of species i.

11. Brillouin index

The Brillouin Index measures biodiversity while accounting for both species abundance and number. The formula is:

$${\text{H}}\_{\text{B }}={\text{ }}\left( {{\text{1 }}/{\text{ N}}} \right){\text{ }}*{\text{ ln }}\left( {{\text{N}}!{\text{ }}/{\text{ }}\prod \left( {{\text{i}}\,=\,{\text{1 to S}}} \right){\text{ n}}\_{\text{i}}!} \right)$$

where N is the total number of individuals, S is the number of species, and n_i is the number of individuals of species i.

12. Berger-parker index

The Berger-Parker Index is a measure of dominance, calculated as the proportion of the most abundant species relative to the total number of individuals:

$${\text{BP}}\,=\,{\text{n}}\_{\text{max }}/{\text{ N}}$$

where n_max is the number of individuals in the most abundant species, and N is the total number of individuals.

13. ACE (Abundance-based Coverage Estimator)

The ACE index estimates the total number of species based on the abundance of rare species. It is calculated using the formula:

$$ACE=S+(n_{1}^{2})/(2{n_2})$$

where S is the observed number of species, n_1 is the number of species represented by one individual, and n_2 is the number of species represented by two individuals.

### Result

**Fig. 1:**
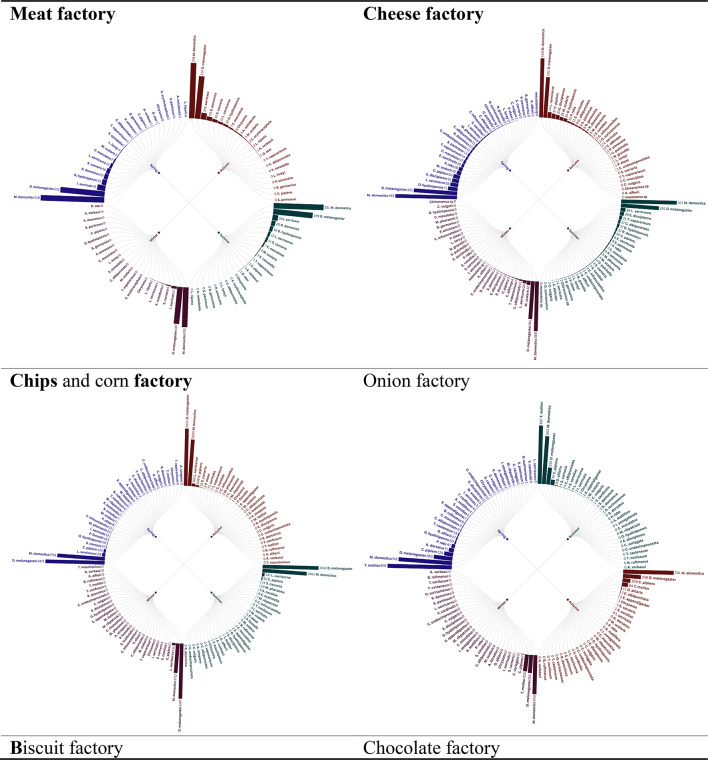

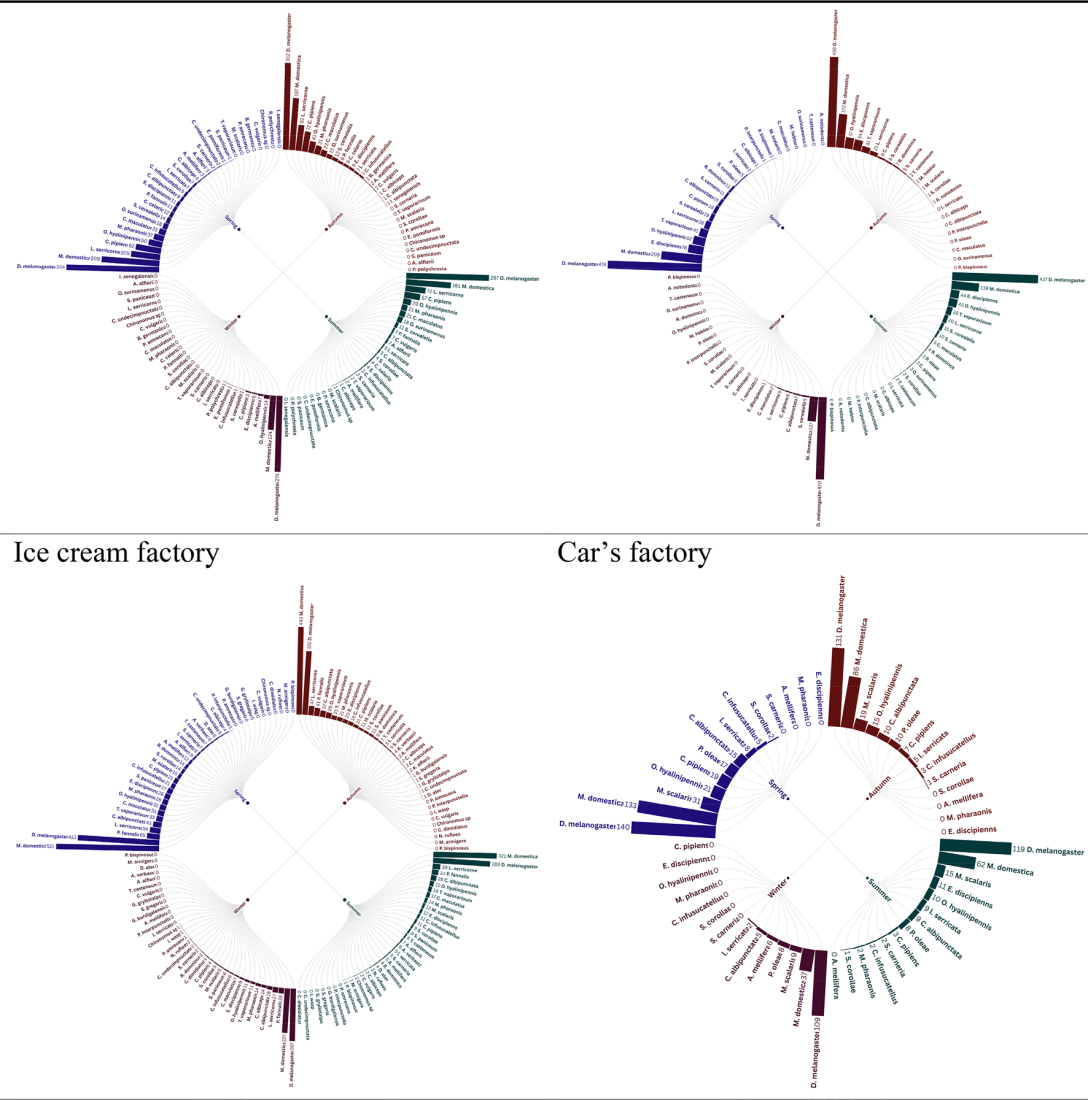
Radial bar tree represents the abundance of insects during 2021 seasons.

**Fig. 2:**
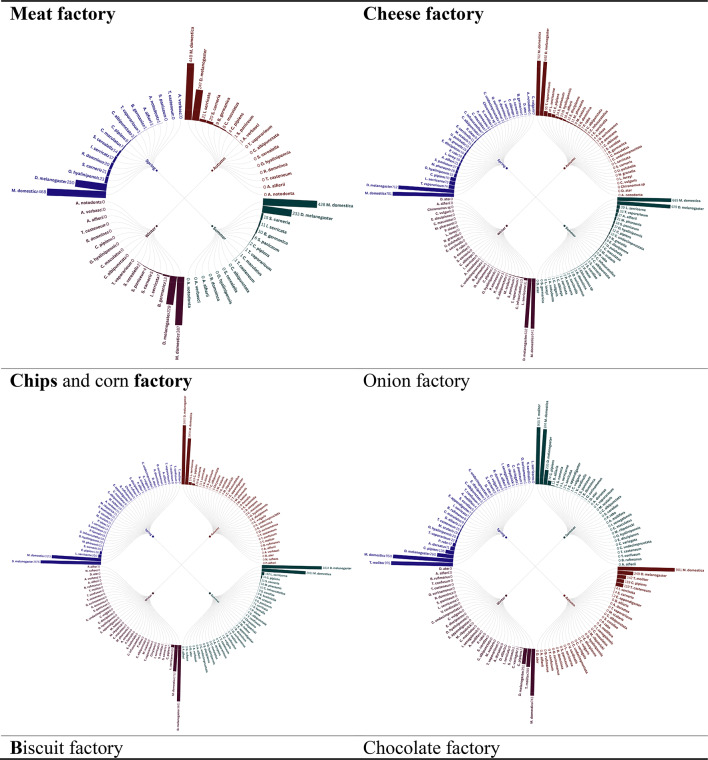

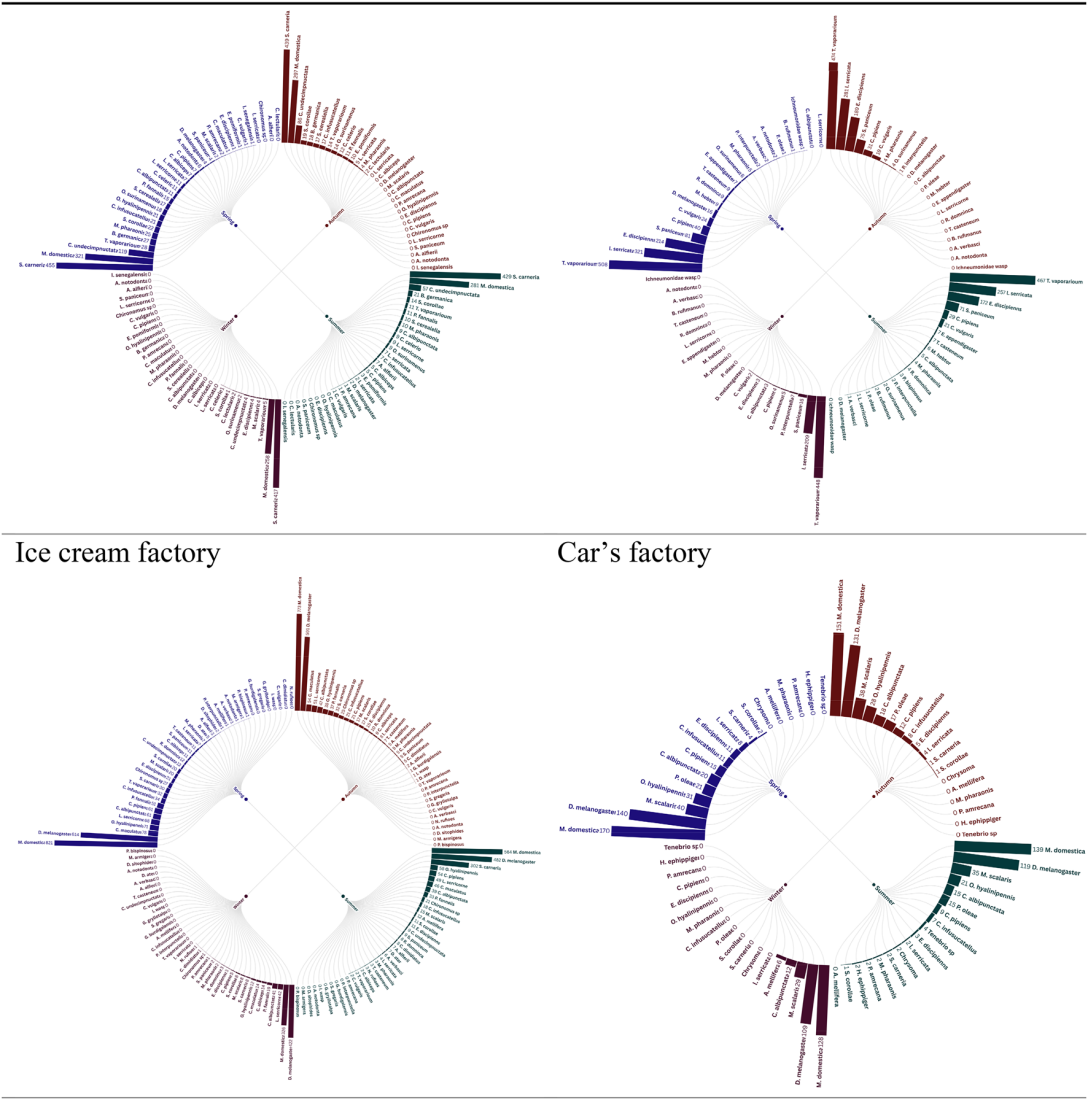
Radial bar tree represents the abundance of insects during 2022 seasons.

Radial bar tree analysis of the trap insect landings in eight factories throughout the seasons 2021 provided clear trends in the abundance and distribution, which is firmly associated with the kind of factory activity. Factories dealing with production of foods, including meat, cheese, chips and corn factories, biscuit, chocolate and ice cream factories recorded increased levels of insect abundance, which was perhaps attributed to presence of organic substances and foods detriments which serve as gourmets to the pests. Conversely, the car factory recorded comparatively smaller numbers of insects since the environmental conditions do not encourage the growth of insects. Interestingly, the onion factory had a distinct insect profile, perhaps due to a distinct set of attractants or repellants that were present with regard to onion processing. The observation shows that factory operations affect the population of insects and that special pest management methods should be developed, depending on the risks presented by the industrial space in question, identical observations held during 2022 seasons (Figs. 1,2).

**Fig. 3:**
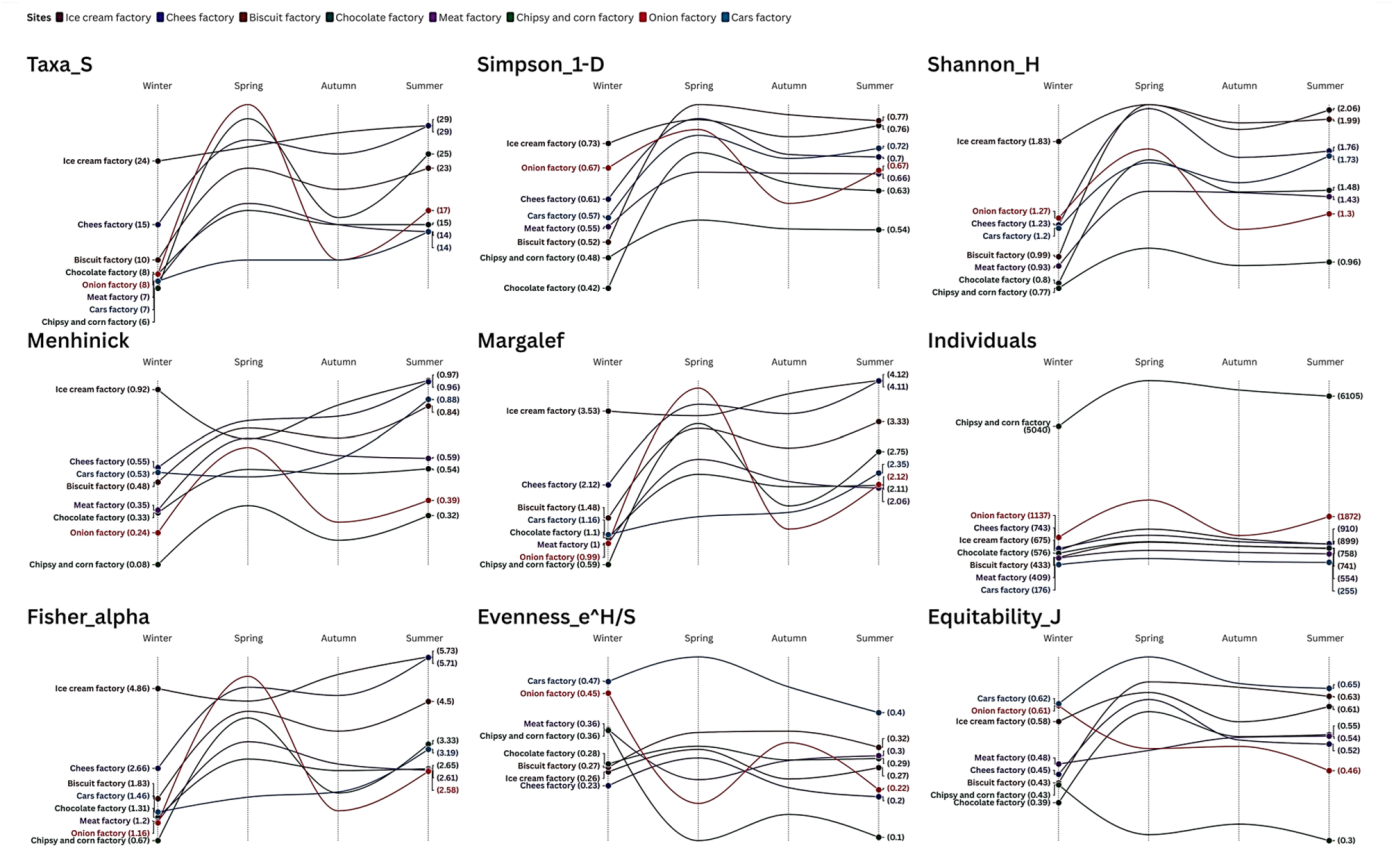
Step slope chart represents calculated environmental indices for studied sites during 2021 seasons.

The comparison of environmental indices in eight factories in 2021 seasons showed a major diversity, dominance, and abundance in insects, which explained factory type and seasonal variations. The Ice Cream Factory had the most taxonomic richness (Taxa_S) and diversity (Shannon_H) all the time, and this could be attributed to the favorable conditions that facilitate insect population growth attributable to the organic refuse in the area. There was also high diversity at the Cheese Factory, but it was lower compared to the Ice Cream Factory. Conversely, the Car Factory was least diverse (Shannon_H = 1.53 ± 0.24) and taxonomically rich (Taxa_S = 10.25 ± 2.87) which indicated that it was not as insect friendly as the other two sites (Table S1, Fig. 3).

The Chips and Corn Factory was found to have the largest population, but the various diversity indices (e.g., Shannon_H = 0.93 ± 0.12) have been found as the lowest indicating that few species dominate the population. This differs with the Onion Factory that was moderately diverse and highly variable between seasons (Table S1 & Fig.  3).

The patterns of dominance were variable with scores on Berger-Parker index segregating high (Chocolate Factory 0.59 ± 0.10) to low dominance (Ice Cream Factory 0.38 ± 0.02), which is indicative of strong dominance by a single species and more balanced community, respectively. There was also seasonality with a tendency towards greater diversity and abundance in spring at most of the sites (Table S1).

Food-based factories (e.g., Ice Cream, Cheese) maintained more enriched and diverse insect communities than those of non-food-based factories (e.g., Car Factory). These results indicate that industrial activity affects insect ecology and that there should be different approaches to pest control, depending on the risks involved in the factory.

**Fig. 4:**
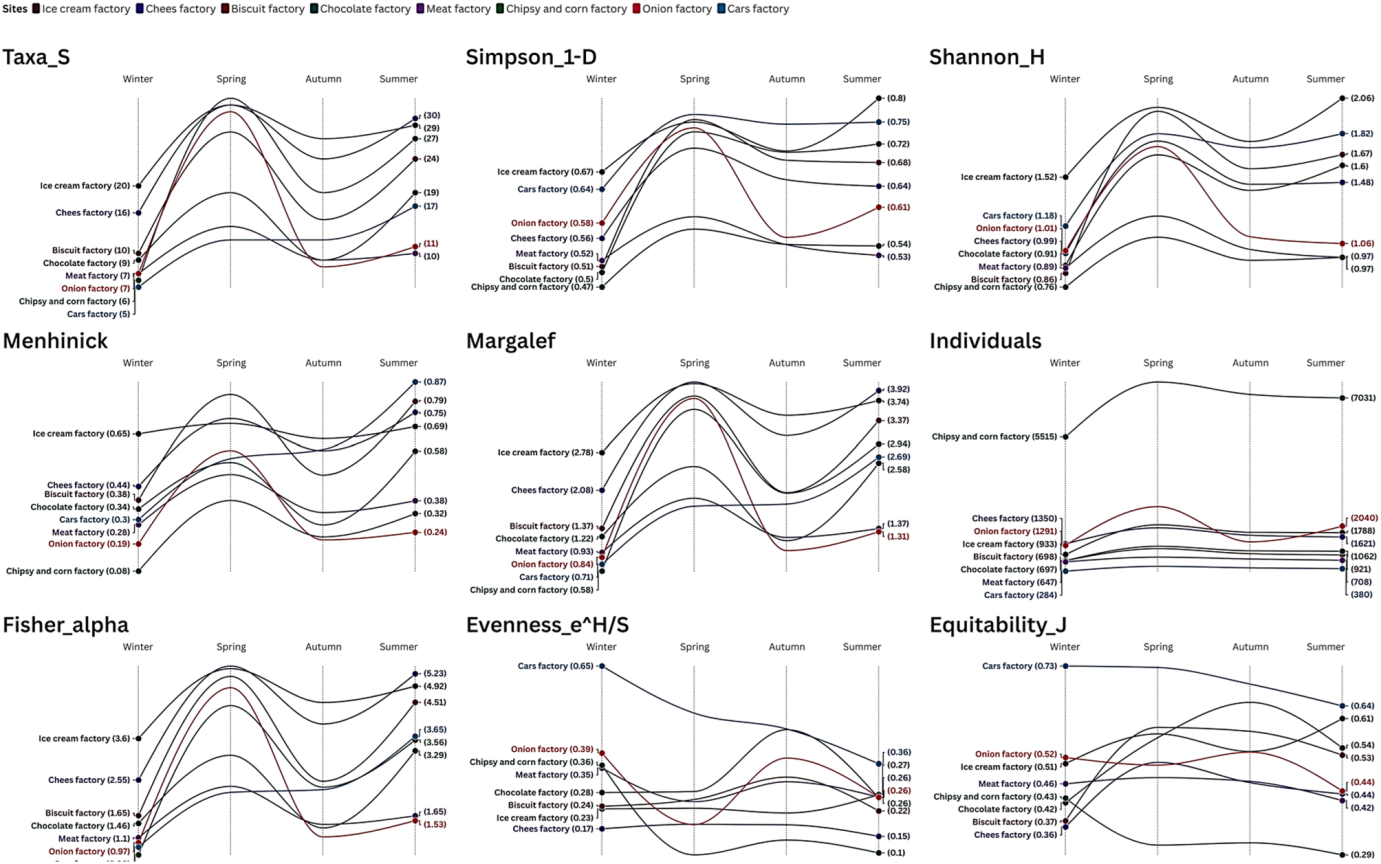
Step slope chart represents calculated environmentalindices for studied sites during 2022 seasons.

The study of the seasonal patterns of the environmental indices of the eight factories showed that insect diversity, dominance, and abundance within eight factories had distinguishable patterns with intriguing fluctuations depending on the type of factory and seasonal changes. The Ice Cream Factory remained the single most biodiverse site with the largest taxonomic richness during the 2022 season (Taxa S, mean = 27 ± 5.1), as well as the Shannon diversity (1.83 ± 0.25) so it was consistently suitable to the insect communities because of the presence of organic wastes. Cheese Factory ranked next with slightly lower diversity (Shannon_H = 1.43 ± 0.32), and the Car s Factory was found to have surprisedly better diversity due to environmental or sampling shifts (Simpson 1-D = 0.73 ± 0.06) in 2022 as compared to 2021 (Table S2, Fig. 4).

The Chips and Corn Factory once more registered the greatest number of individuals during 2022 season (6843.75 ± 925.52), but the diversity indices of the samples (e.g. Shannon_H = 0.95 ± 0.14) showed signs of remaining dominated by only few species. By contrast, the Onion Factory exhibited wild variability, with peaks of diversity in the spring coupled with precipitous drops at other times, presumably because of the repellent qualities of onion products (Table S2 & Fig. 4).

The intensity of dominance patterns was also strong in the Meat Factory during 2022 season (Berger-Parker = 0.59 ± 0.02) and Chips and Corn Factory (0.56 ± 0.07) and dominant species dominance and the Ice Cream Factory was the most balanced community (Berger-Parker = 0.4 ± 0.06). The major seasonal modes exhibited in diversity were spring in most sites, where most insect activities transpired (Table S2).

Interestingly, the Cars Factory had greater evenness during 2022 (Equitability_J = 0.7 ± 0.04) than food-related factories implying that species distribution across it may be more even though species richness is lower. These results support the argument that industrial activity has significant role to play in shaping insect ecology as food-processing factories have more varied but uneven communities than non-food sites. The findings identify a requirement in strategically moving towards seasonally adaptive pest management policies dependent on particular factory settings (Table S2 & Fig. 4).

**Fig. 5:**
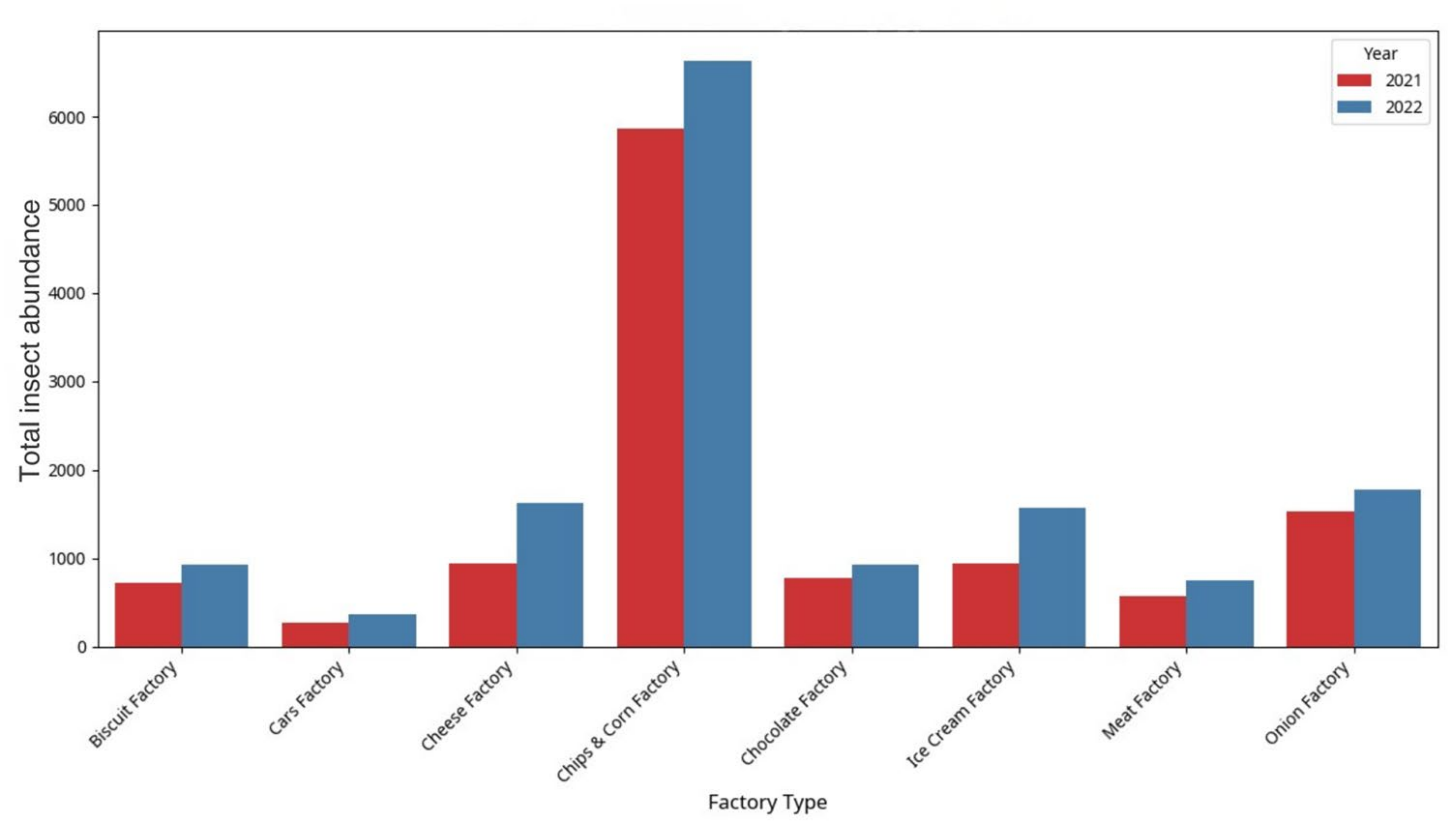
Total insect abundance by factory type and year.

Total insect abundance between the factories and seasons (2021 and 2022) is subject to considerable differences (*P* < 0.05), while clearly tends toward chips & corn factory. As shown in Figure (5), manifold numbers of insects are found always dominating in some types of factories than in others. Importantly, the greatest total insect abundance in 2021 and 2022 is observed in the chips & corn factory with its abundance rising significantly in 2022, The Chips & Corn Factory has the highest total abundance by a wide margin (12,499.30), which is almost half of the total abundance recorded. The Onion Factory (3,302.10), the Cheese Factory (2563.50), and the Ice Cream Factory (2,523.60) come next. The Cars Factory shows the lowest abundance (626.20), which is expected given its non-food-related operations.

Other types of the factory like cheese factory and ice cream factory also demonstrate significant numbers of insects (*P* < 0.05) including an observable increment in the year 2022 as compared to the year 2021. Such an incidence of greater abundance of insects in 2022 is relatively similar with most factory types.

On the contrary, the lowest abundance of insects is continuously experienced in cars factory in both years, and this is understandable due to the industrial nature of the factory and the probable limited resources available to insect populations. The biscuit factory, chocolate factory and meat factory report moderate levels of abundance of insects, and fluctuations exist between the two years (Fig. 5).

**Fig. 6:**
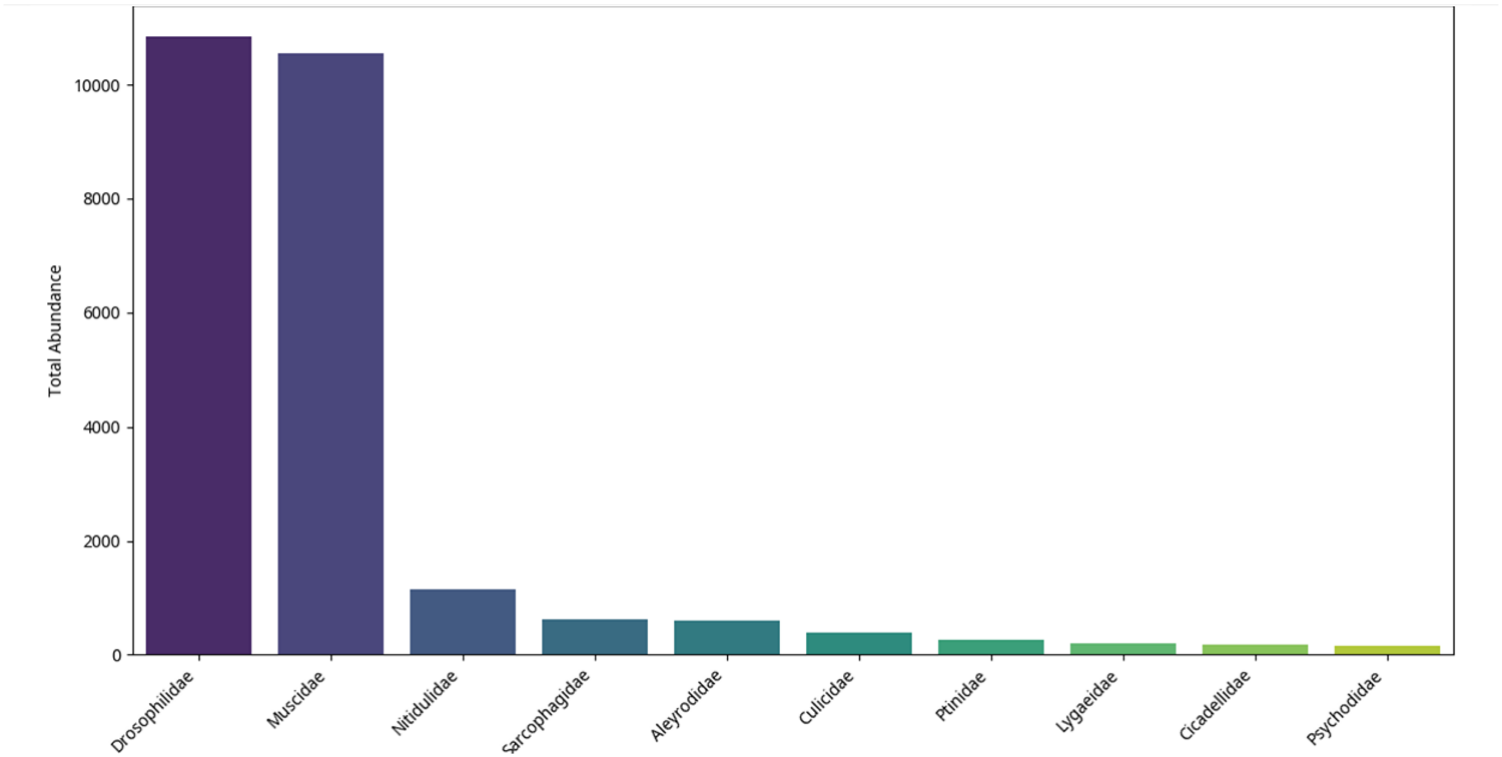
Top 10 Insect families by abundance.

The total abundance analysis of insect families helps to understand which are the most common ones among all types of factories and in all seasons. Figure (6) shows that Drosophilidae and Muscidae are preponderant and are very much ahead of other families. Since the two families are largely attributed to human habitats and astrophysical resources. Fruit flies (Drosophilidae) tend to colonize those regions that contain fermented fruits or dead biological material, which may be concentrated on different levels in the types of factories and those related to food products. These flies, such as common house flies (Muscidae), are also everywhere and popularize in an environment where organic trash or food leftovers are available. The fact that they are highly concentrated implies that there might be some issues with sanitation and bug control in such factory premises.

Next are Nitidulidae (sap beetles) and Sarcophagidae (flesh flies) which have moderate representation after these two predominant families (*P* < 0.05). Nitidulidae are usually also drawn to rotting plants or overripe fruits, whereas the Sarcophagidae are scavengers and tend to be found near decaying animal matters. Their occurrence, not as plentiful as those of Drosophilidae and Muscidae, still shows the presence of breeding and feeding conditions in as well as about the factories (Figure, 6).

Other families such as Aleyrodidae (whiteflies), Culicidae (mosquitoes), Ptinidae (spider beetles), Lygaeidae (seed bugs), Cicadellidae (leafhoppers) and Psychodidae (moth flies) also occur in significantly smaller numbers. Aleyrodidae may indicate plant material close to the factories and Culicidae standing water sources. The related moths infest food-related factories including Ptinidae that might infest the stored products. Their low abundance indicates that these families do not have good conditions to reproduce as compared to Drosophilidae and Muscidae (Fig. 6).

**Fig. 7:**
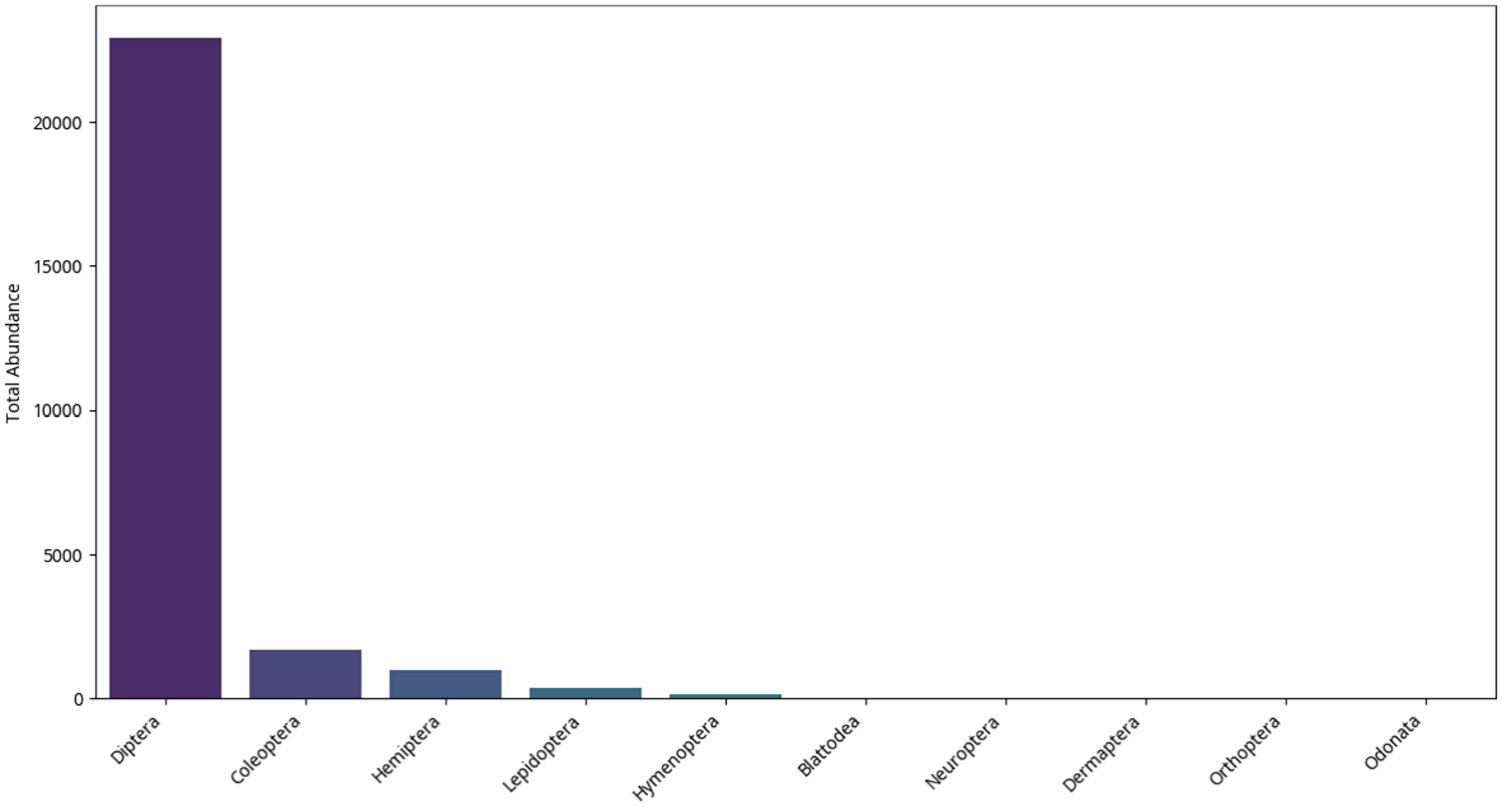
Top 10 insect orders by abundance.

Figure 7 represents the most dominant orders of all the types of factories and through seasons. Within diptera (true flies) by far the most abundant insect order (*P* < 0.05), this finding is in agreement with the predominance of the Muscidae (house flies) and Drosophilidae (fruit flies) families (which belong to Diptera). This helps to bring out the fact that flies as a whole are the most common within these factory settings. In particular in the chips & corn factory.

Mosquitoes are also common (*P* < 0.05) particularly in the chips & corn factory as well as in the biscuit factory. Coleoptera (Beetles) is second largest in number after Diptera. Spider beetles, Ptinidae are abundant in the ice cream factory (33.3 ± 12.5 in 2021, 42.5 ± 11.1 in 2022) and chocolate factory (60.8 ± 34.9 in 2022) (Fig. 7).

Sap beetles (Nitidulidae) also have a very high number (*P* < 0.05) in the onion factory (553.8 ± 381.9 in 2021, 576.8 ± 381.9 in 2022), which may indicate strong attraction to the environment of onions. Moths and butterflies (Lepidoptera) Pyralidae and Crambidae Food related factories (e.g. Ice cream, biscuit). Pieridae (butterflies) are mainly present in the cheese factory although adults of moths and butterflies may not necessarily present a contamination risk their larval stages (caterpillars) may be important pests mostly in the factory handling stored products or plants. Their occurrence implies requiring adult and larval monitoring and control (Fig. 7).

True bugs (Hemiptera, aphids and cicadas) are also abundant. There are Hemipterans, by family depending on certain families, which may be related to plant material or outdoor environments at large (Fig. 7).

Others, such as Hymenoptera (ants, bees, and wasps), Blattodea (cockroaches), Orthoptera (grasshoppers, crickets), Psocoptera (booklice), Thysanoptera (thrips) and Neuroptera (lacewings) are found in much lower levels (*P* < 0.05). Occurrence of Blattodea is a hygienic worry, even in small quantities, as they are the notorious disease transmitters. Hymenoptera could entail ants that are magnetized toward food. The fact that the rest of the orders are very small would indicate that they are either casual visitors to the places or that the factory locations are not very favorable to the respective populations.

As it is depicted, there was a higher insect population in 2022 compared to 2021, especially among Diptera and Coleoptera. The number of Muscidae increased in the meat factory by 262 (more than five times) to 433 in 2022. In the same way, the number of Drosophilidae in the cheese factory rose to 678.8 ± 32.5 as compared to 296 ± 19.1 Diptera, in 2022.

The Hymenoptera like the Formicidae (ants) fluctuated but were relatively maintainable and more so at the ice cream and biscuit factories. Cimicidae (bed bugs) were absent altogether excepting 1 random time in 2022 at the biscuit factory. chips & corn factories are considered one of the most infested factories, particularly with Muscidae and Drosophilidae The reason is given that moisture is high and there is a lot of starch. Onion Factory, Nitidulidae population recorded as very high, this could be because of the exorbitant smell and the build-up of organic materials. Surprisingly cars factory had a significant absence of insects (*P* < 0.05), there were Plutellidae (diamondback moths) and Tenebrionidae (darkling beetles). Probably the result of externally conducive factors in the environment and not internal pollution. Ice cream and cheese processing contain high numbers of pest species Drosophilidae, Muscidae, and Lepidoptera, Suggests difficulties in dealing with perishables and fermenting substrates.

## Discussion

The Radial bar tree analysis can be corroborated to accepted ecological concepts, where the increasing abundance of Food-Processing Factories, High abundances in meat, dairy, and starch rich facilities (e.g., chips, ice cream) is indicative of the concept of Resource Concentration Hypothesis^15^, which holds that high densities of organic waste result in hot spots of saprophagous Diptera (e.g., Muscidae) and Lepidoptera (e.g., Pyralidae). These findings confirm Integrated Pest Management (IPM) programs specific to industrial niche food factories target Diptera with pheromone traps^16^ and sanitation techniques.

Specific Profile of onion factory produces volatile compounds (e.g. thiosulfinates) that repel generalist insects but also could have specialized pests (e.g. *Delia antiqua*, the onion fly). Car Factories Low Abundance, The sterile environment composed mainly of metals does not include organic substrates, as it is in keeping with the Habitat Filtering Concept^17^.

Significant seasonal contrast in diversity of species was observed where high diversity indices between seasons were found in the study. Which indicates an environmental deterministic process rather than a random one. The highest abundance in warmer seasons can be related to the Thermal Performance Curve^18^ wherein insects have their highest metabolic rates at intermediate temperatures. Muscidflies and Drosophilidae are dominant in decaying matter as expected^19^. There are 93 distinct insect species that were found in the course of the present research. All of these species were divided into ten orders and 55 families.

High Diversity in Food-Processing Factories Taxonomic richness (Taxa_S) and Shannon diversity (H) were high in the two ice cream and cheese factories^20^. Organic residues (sugars, fats) open sinecure positions to various taxa, e.g.: Lepidoptera (e.g., Pyralidae) snack on sugary substrates^21^. Diptera (e.g. Drosophilidae) which grow in fermenting organic matter^22^. Low Diversity, High Abundance at chips & corn factory, the near-competitive exclusion of abundance (*N* = 6030 ± 698) and low diversity (H = 0.93) are consistent with this seemingly high nutrient status provided by starches/oils and with Reduced niche partitioning^23^.

Spring has its greatest diversity coupled up with temperature-dependent activity^18^ and resource pulse^24^. Diptera make use of warmer climates to breed fast, while Lepidoptera timing the larval emergence with plant/floral debris. This match has a high Berger Parker index, clearly monodominant, probably with a stored product pest^25^.

Overall increase in Diptera (e.g., Muscidae, Drosophilidae) and Coleoptera between 2021 and 2022 indicates such an impact on population density and resource availability. Temperature & Humidity, Warmer temperatures make the lifecycle of insects faster (e.g., growth time of Muscidae become faster at higher temperatures)^18^. Breeding sites could have been also enhanced by increased production of organic waste (e.g. Meat Factory). Lapse in Pest Control, Decreased effectiveness of IPM practices might be the reason behind rush (e.g., Drosophilidae in cheese factory). Stark swings in Particular Taxa, Hemiptera (Aleyrodidae) in chocolate factory, this is probably attributed towards enhancing the plant debris or new attack of host plants. Sugary excretions are utilized by whiteflies (Aleyrodidae)^26^.

Coleoptera (Ptinidae) Collapse in biscuit factory may be indicative of better sanitation or the use of pesticides against stored-product pests. Ants (Formicidae) are generalist foragers with stable colonies that are buffered against daily environmental fluctuations^27^. Their occurrence in ice cream/biscuit Factories indicates that there remain residues of sugar/proteins. Cimicidae (Bed Bugs) Only one instance in biscuit factory during 2022 probably an accidental introduction (e.g. on packaging). Bed bugs only feed on human beings, so factories are an unlikely habitat choice^28^.

Chips and corn factory characterized by fermentation odor results in volatiles which attract flies to starch^22^. The larval development occurs within moisture^29^. Cars Factory, surprise findings of insects Plutellidae (Diamondback Moths) and Tenebrionidae Possibly short-term colonies of surrounding plants^30^.

The prevalence of Muscidae and Drosophilidae species was observed as a result of the fast reproduction and the liking to decaying organic matter^22^. Coleoptera (15% of total abundance) represented in Ptinidae (spider beetles) in ice cream/chocolate factories point to indicator of stored-product infestations^31^. Outbreaks of Nitidulidae are indicators of bad waste management.

## Conclusion

The two-year comparative analysis of the population of insects in different industrial conditions at 6th October City provides compelling data supporting the significant influence of factory type and industrial-related aspects of activity thus affecting local entomological ecology. The regularities, which might be particularly the high diversity and richness of insects in food-processing surroundings (such as ice cream and cheese factories) and low diversity in non-food environments (such as car factory), indicate the fundamental ecological concept that the primary factors, which affect the structure of communities, are the suitability of the habitat and availability of resources. The findings of the study and, in particular, the ongoing dominance of Diptera (Muscidae and Drosophilidae) and the influence of the factors associated with food resources underscore the role of special and evolutional approaches to the control of pests in the industrial environment. The observed changes, including the unexpected number of different species at the Car Factory and the random trends of the onion factory focus on the complexity in the play of the environmental situations, and the facilities of resource endowment, and the adapting tendencies of species. The presented findings play a pivotal role in long-term and effective pest management, as integrating the laws of biology governing the organization of insect communities with unique peculiarities of an industrial atmosphere are supposed to help gain long-term and effective pest management strategies. Further research in the area of species level, microhabitat studies, and extended monitoring will allow a better understanding and result in more accurate and ecologically sound pest management measures. Officials responsible for these food industries should apply the highest standards of Integrated Pest Management (IPM) within production area and hygiene and warehouses inside the factory. The presence of this large number of insects within these high-risk areas indicates that the insects are capable of entering these areas through entry points such as doors, windows, and others, as the foodstuffs inside these factories are strong attractants for insects. It must also be taken into consideration that the threat to food industries is not only from house flies, but also that Drosophila and other insect species from other orders were strongly present. Searching for the breeding sources of these insects whether inside the factory within production devices, and raw materials, or outside in the vicinity of buildings, fences, internal plantings, and even outside the fences for a sufficient distance and external plantings are effective ways to reduce food contamination and maintain food safety. The results of this study enable us to prepare a proactive plan to control each type of insect that was mentioned. This plan is related to the seasons of its appearance to avert the risk of its population increasing, which would make control operations more difficult. Crucially, the proactive preventative plan saves on the use of pesticides and reduces the accidental contamination of the final product and raw materials with pesticides.

## Supplementary Information

Below is the link to the electronic supplementary material.

Supplementary Material 1

## Data Availability

The datasets used and/or analyzed during the current study are available from the corresponding author on reasonable request.
