# Supplementary material for: Influence of different industrial resource profiles on taxonomical richness and community structure of insects populations
Source: Sci Rep. 2026 Jan 6;16:627. doi: 10.1038/s41598-025-32865-3 (PMC12774964; doi:10.1038/s41598-025-32865-3)
Supplement: Supplementary file 1 — Supplementary Material 1 [file 41598_2025_32865_MOESM1_ESM.docx]

**Influence of Different Industrial Resource Profiles on Taxonomical Richness and Community Structure of Insects populations**

**Mohamed A. M. Shahat^1^, Mohamed A.M. El-Tabakh^1*^, Yasser I. Hamza^1^, Ahmed M. A. Elnaggar^1^, Wesam M.A. Ward^1^, Heba F. Abd-Elkhalek^2^, Ahmed Z.I. Shehata^1^**

1. **Zoology Department, Faculty of Science, Al-Azhar University, Cairo, Egypt**
2. **Entomology Department, Faculty of Science, Benha University, Benha, Egypt.**

**Corresponding author:** Mohamed A.M. El-Tabakh**; Email:** [dr.m.eltabakh.201@azhar.edu.eg](mailto:dr.m.eltabakh.201@azhar.edu.eg)

**Table (S1):** Calculated Environmental indices for studied sites during 2021 season

| indices | Sites | Winter | Spring | Autumn | Summer | Mean±SD |
| --- | --- | --- | --- | --- | --- | --- |
| Taxa_S | Ice cream factory | 24 | 26 | 28 | 29 | 26.75 ± 2.22a |
|  | Cheese factory | 15 | 27 | 25 | 29 | 24 ± 6.22a,b |
|  | Biscuit factory | 10 | 23 | 20 | 23 | 19 ± 6.16b,c |
|  | Chocolate factory | 8 | 17 | 15 | 15 | 13.75 ± 3.95c,d |
|  | Meat factory | 7 | 18 | 15 | 14 | 13.5 ± 4.65c,d |
|  | Chips and corn factory | 6 | 30 | 16 | 25 | 19.25 ± 10.56b,c |
|  | Onion factory | 8 | 32 | 10 | 17 | 16.75 ± 10.87c |
|  | Car’s factory | 7 | 10 | 10 | 14 | 10.25 ± 2.87d |
| Simpson_1-D | Ice cream factory | 0.7254 | 0.7754 | 0.7394 | 0.7631 | 0.75 ± 0.02a |
|  | Cheese factory | 0.6078 | 0.778 | 0.7023 | 0.6969 | 0.7 ± 0.07a,b,c |
|  | Biscuit factory | 0.5172 | 0.8075 | 0.786 | 0.7734 | 0.72 ± 0.14b,c,d |
|  | Chocolate factory | 0.4199 | 0.7062 | 0.642 | 0.6255 | 0.6 ± 0.12d,e |
|  | Meat factory | 0.5497 | 0.6646 | 0.6625 | 0.6611 | 0.63 ± 0.06c,d |
|  | Chips and corn factory | 0.4842 | 0.5638 | 0.5452 | 0.5431 | 0.53 ± 0.03e |
|  | Onion factory | 0.6737 | 0.7548 | 0.5988 | 0.6685 | 0.67 ± 0.06b,c,d |
|  | Car’s factory | 0.5687 | 0.7425 | 0.6937 | 0.7152 | 0.68 ± 0.08a,b,c |
| Shannon_H | Ice cream factory | 1.83 | 2.098 | 1.917 | 2.058 | 1.98 ± 0.12a |
|  | Cheese factory | 1.225 | 2.068 | 1.715 | 1.761 | 1.69 ± 0.35b |
|  | Biscuit factory | 0.9948 | 2.097 | 1.965 | 1.991 | 1.76 ± 0.51a,b |
|  | Chocolate factory | 0.8022 | 1.697 | 1.464 | 1.476 | 1.36 ± 0.39c |
|  | Meat factory | 0.9271 | 1.469 | 1.459 | 1.43 | 1.32 ± 0.26c |
|  | Chips and corn factory | 0.7659 | 1.057 | 0.9303 | 0.9557 | 0.93 ± 0.12d |
|  | Onion factory | 1.275 | 1.776 | 1.192 | 1.305 | 1.39 ± 0.26c |
|  | Car’s factory | 1.201 | 1.675 | 1.531 | 1.725 | 1.53 ± 0.24b,c |
| Menhinick | Ice cream factory | 0.9238 | 0.6883 | 0.8497 | 0.9672 | 0.86 ± 0.12a |
|  | Cheese factory | 0.5503 | 0.7756 | 0.7966 | 0.9613 | 0.77 ± 0.17a,b |
|  | Biscuit factory | 0.4806 | 0.74 | 0.6921 | 0.8449 | 0.69 ± 0.15b,c |
|  | Chocolate factory | 0.3333 | 0.5417 | 0.5207 | 0.5448 | 0.49 ± 0.1d,e |
|  | Meat factory | 0.3461 | 0.6913 | 0.6083 | 0.5948 | 0.56 ± 0.15c,d |
|  | Chips and corn factory | 0.08452 | 0.3677 | 0.2012 | 0.32 | 0.24 ± 0.13f |
|  | Onion factory | 0.2373 | 0.6461 | 0.2883 | 0.3929 | 0.39 ± 0.18e,f |
|  | Car’s factory | 0.5276 | 0.5057 | 0.5903 | 0.8767 | 0.63 ± 0.17b,c,d |
| Margalef | Ice cream factory | 3.53 | 3.442 | 3.863 | 4.117 | 3.74 ± 0.31a |
|  | Cheese factory | 2.118 | 3.662 | 3.482 | 4.11 | 3.34 ± 0.86a,b |
|  | Biscuit factory | 1.483 | 3.201 | 2.824 | 3.329 | 2.71 ± 0.85b,c |
|  | Chocolate factory | 1.101 | 2.321 | 2.083 | 2.111 | 1.9 ± 0.55d |
|  | Meat factory | 0.9977 | 2.608 | 2.184 | 2.058 | 1.96 ± 0.68c,d |
|  | Chips and corn factory | 0.5865 | 3.294 | 1.714 | 2.753 | 2.09 ± 1.2c,d |
|  | Onion factory | 0.9949 | 3.972 | 1.269 | 2.123 | 2.09 ± 1.34c,d |
|  | Car’s factory | 1.16 | 1.508 | 1.59 | 2.346 | 1.65 ± 0.5d |
| Individuals | Ice cream factory | 675 | 1427 | 1086 | 899 | 1021.75 ± 318.15c |
|  | Cheese factory | 743 | 1212 | 985 | 910 | 962.5 ± 194.67c |
|  | Biscuit factory | 433 | 966 | 835 | 741 | 743.75 ± 226.79c,d |
|  | Chocolate factory | 576 | 985 | 830 | 758 | 787.25 ± 169.72c,d |
|  | Meat factory | 409 | 678 | 608 | 554 | 562.25 ± 114.08d,e |
|  | Chips and corn factory | 5040 | 6655 | 6321 | 6105 | 6030.25 ± 697.86a |
|  | Onion factory | 1137 | 2453 | 1203 | 1872 | 1666.25 ± 620.76b |
|  | Car’s factory | 176 | 391 | 287 | 255 | 277.25 ± 89.03e |
| Fisher_alpha | Ice cream factory | 4.857 | 4.514 | 5.246 | 5.729 | 5.09 ± 0.52a |
|  | Cheese factory | 2.662 | 4.895 | 4.667 | 5.712 | 4.48 ± 1.29a,b |
|  | Biscuit factory | 1.827 | 4.232 | 3.685 | 4.501 | 3.56 ± 1.2b,c |
|  | Chocolate factory | 1.315 | 2.919 | 2.6 | 2.65 | 2.37 ± 0.72d |
|  | Meat factory | 1.2 | 3.395 | 2.782 | 2.611 | 2.5 ± 0.93c,d |
|  | Chips and corn factory | 0.6725 | 4.051 | 1.983 | 3.326 | 2.51 ± 1.49c,d |
|  | Onion factory | 1.162 | 5.195 | 1.494 | 2.58 | 2.61 ± 1.83c,d |
|  | Car’s factory | 1.458 | 1.87 | 2.013 | 3.185 | 2.13 ± 0.74d |
| Evenness_e^H/S | Ice cream factory | 0.2597 | 0.3134 | 0.243 | 0.2699 | 0.27 ± 0.03b,c |
|  | Cheese factory | 0.2268 | 0.293 | 0.2222 | 0.2007 | 0.24 ± 0.04b,c |
|  | Biscuit factory | 0.2704 | 0.3539 | 0.3568 | 0.3183 | 0.32 ± 0.04b |
|  | Chocolate factory | 0.2788 | 0.321 | 0.2883 | 0.2917 | 0.29 ± 0.02b |
|  | Meat factory | 0.361 | 0.2413 | 0.2868 | 0.2983 | 0.3 ± 0.05b |
|  | Chips and corn factory | 0.3585 | 0.09596 | 0.1585 | 0.104 | 0.18 ± 0.12c |
|  | Onion factory | 0.4473 | 0.1847 | 0.3295 | 0.217 | 0.29 ± 0.12b |
|  | Car’s factory | 0.475 | 0.5338 | 0.4624 | 0.4009 | 0.47 ± 0.05a |
| Equitability_J | Ice cream factory | 0.5758 | 0.6439 | 0.5754 | 0.6111 | 0.6 ± 0.03a,b |
|  | Cheese factory | 0.4522 | 0.6275 | 0.5327 | 0.523 | 0.53 ± 0.07b |
|  | Biscuit factory | 0.432 | 0.6687 | 0.656 | 0.6349 | 0.6 ± 0.11a,b |
|  | Chocolate factory | 0.3858 | 0.599 | 0.5407 | 0.5451 | 0.52 ± 0.09b |
|  | Meat factory | 0.4764 | 0.5082 | 0.5387 | 0.5417 | 0.52 ± 0.03b |
|  | Chips and corn factory | 0.4275 | 0.3109 | 0.3355 | 0.2969 | 0.34 ± 0.06c |
|  | Onion factory | 0.6131 | 0.5126 | 0.5178 | 0.4607 | 0.53 ± 0.06b |
|  | Car’s factory | 0.6174 | 0.7273 | 0.665 | 0.6536 | 0.67 ± 0.05a |
| Dominance_D | Ice cream factory | 0.2746 | 0.2246 | 0.2606 | 0.2369 | 0.25 ± 0.02e |
|  | Cheese factory | 0.3922 | 0.222 | 0.2977 | 0.3031 | 0.3 ± 0.07c,d,e |
|  | Biscuit factory | 0.4828 | 0.1925 | 0.214 | 0.2266 | 0.28 ± 0.14d,e |
|  | Chocolate factory | 0.5801 | 0.2938 | 0.358 | 0.3745 | 0.4 ± 0.12a,b |
|  | Meat factory | 0.4503 | 0.3354 | 0.3375 | 0.3389 | 0.37 ± 0.06b,c |
|  | Chips and corn factory | 0.5158 | 0.4362 | 0.4548 | 0.4569 | 0.47 ± 0.03a |
|  | Onion factory | 0.3263 | 0.2452 | 0.4012 | 0.3315 | 0.33 ± 0.06b,c,d |
|  | Car’s factory | 0.4313 | 0.2575 | 0.3063 | 0.2848 | 0.32 ± 0.08c,d,e |
| Brillouin | Ice cream factory | 1.75 | 2.049 | 1.857 | 1.981 | 1.91 ± 0.13a |
|  | Cheese factory | 1.183 | 2.013 | 1.658 | 1.69 | 1.64 ± 0.34b,c |
|  | Biscuit factory | 0.9504 | 2.038 | 1.908 | 1.918 | 1.7 ± 0.51a,b |
|  | Chocolate factory | 0.7737 | 1.655 | 1.423 | 1.429 | 1.32 ± 0.38d |
|  | Meat factory | 0.8943 | 1.414 | 1.404 | 1.375 | 1.27 ± 0.25d |
|  | Chips and corn factory | 0.763 | 1.047 | 0.9244 | 0.9464 | 0.92 ± 0.12e |
|  | Onion factory | 1.258 | 1.747 | 1.173 | 1.286 | 1.37 ± 0.26d |
|  | Car’s factory | 1.12 | 1.612 | 1.454 | 1.61 | 1.45 ± 0.23c,d |
| Berger-Parker | Ice cream factory | 0.3956 | 0.3651 | 0.4079 | 0.3571 | 0.38 ± 0.02d |
|  | Cheese factory | 0.4966 | 0.3754 | 0.4457 | 0.4516 | 0.44 ± 0.05c,d |
|  | Biscuit factory | 0.6328 | 0.3458 | 0.3737 | 0.4008 | 0.44 ± 0.13c,d |
|  | Chocolate factory | 0.7292 | 0.4853 | 0.5518 | 0.5765 | 0.59 ± 0.1a |
|  | Meat factory | 0.4914 | 0.4705 | 0.4572 | 0.4531 | 0.47 ± 0.02b,c |
|  | Chips and corn factory | 0.6323 | 0.514 | 0.5222 | 0.5294 | 0.55 ± 0.06a,b |
|  | Onion factory | 0.4776 | 0.3632 | 0.5827 | 0.4311 | 0.46 ± 0.09c,d |
|  | Car’s factory | 0.6193 | 0.3581 | 0.4564 | 0.4667 | 0.48 ± 0.11b,c |
| ACE | Ice cream factory | 25.44 | 26.19 | 32.54 | 29.76 | 28.48 ± 3.3a |
|  | Cheese factory | 17.16 | 27.94 | 29.39 | 29.86 | 26.09 ± 6.01a,b |
|  | Biscuit factory | 15.05 | 24.11 | 22.18 | 23.72 | 21.27 ± 4.23a,b,c |
|  | Chocolate factory | 8.368 | 20.38 | 17.28 | 15 | 15.26 ± 5.09c,d |
|  | Meat factory | 9.612 | 32.94 | 16.33 | 20.43 | 19.83 ± 9.81b,c |
|  | Chips and corn factory | 7.033 | 31.86 | 18 | 26.9 | 20.95 ± 10.91b,c |
|  | Onion factory | 8.571 | 36.61 | 17.42 | 21.79 | 21.1 ± 11.71b,c |
|  | Car’s factory | 7 | 10 | 10.39 | 14.6 | 10.5 ± 3.13c,d |

*Means that do not share a letter are significantly different.*

**Table (S2):** Calculated Environmental indices for studied sites during 2022 season

| indices | Sites | Winter | Spring | Autumn | Summer | Mean±SD |
| --- | --- | --- | --- | --- | --- | --- |
| Taxa_S | Ice cream factory | 20 | 32 | 27 | 29 | 27 ± 5.1a |
|  | Cheese factory | 16 | 32 | 24 | 30 | 25.5 ± 7.19a,b |
|  | Biscuit factory | 10 | 28 | 15 | 24 | 19.25 ± 8.22b,c |
|  | Chocolate factory | 9 | 19 | 9 | 19 | 14 ± 5.77c,d |
|  | Meat factory | 7 | 14 | 9 | 10 | 10 ± 2.94d |
|  | Chips and corn factory | 6 | 33 | 19 | 27 | 21.25 ± 11.67a,b |
|  | Onion factory | 7 | 31 | 8 | 11 | 14.25 ± 11.3c,d |
|  | Car’s factory | 5 | 12 | 12 | 17 | 11.5 ± 4.93d |
| Simpson_1-D | Ice cream factory | 0.6688 | 0.7542 | 0.7047 | 0.795 | 0.73 ± 0.06a |
|  | Cheese factory | 0.5551 | 0.7096 | 0.6554 | 0.6444 | 0.64 ± 0.06b |
|  | Biscuit factory | 0.507 | 0.7585 | 0.6889 | 0.6849 | 0.66 ± 0.11b |
|  | Chocolate factory | 0.4969 | 0.7376 | 0.7026 | 0.7167 | 0.66 ± 0.11b |
|  | Meat factory | 0.5171 | 0.5922 | 0.5443 | 0.526 | 0.54 ± 0.03c |
|  | Chips and corn factory | 0.4717 | 0.571 | 0.5449 | 0.5421 | 0.53 ± 0.04c |
|  | Onion factory | 0.5813 | 0.7443 | 0.5568 | 0.6084 | 0.62 ± 0.08b |
|  | Car’s factory | 0.6392 | 0.7672 | 0.7506 | 0.7542 | 0.73 ± 0.06a |
| Shannon_H | Ice cream factory | 1.518 | 1.997 | 1.763 | 2.06 | 1.83 ± 0.25a |
|  | Cheese factory | 0.9946 | 1.766 | 1.469 | 1.482 | 1.43 ± 0.32b,c,d |
|  | Biscuit factory | 0.8585 | 1.97 | 1.576 | 1.673 | 1.52 ± 0.47b,c |
|  | Chocolate factory | 0.9126 | 1.671 | 1.427 | 1.599 | 1.4 ± 0.34c,d |
|  | Meat factory | 0.894 | 1.251 | 1.022 | 0.9677 | 1.03 ± 0.15e,f |
|  | Chips and corn factory | 0.7632 | 1.106 | 0.9476 | 0.9674 | 0.95 ± 0.14f |
|  | Onion factory | 1.013 | 1.728 | 1.109 | 1.062 | 1.23 ± 0.34d,e |
|  | Car’s factory | 1.182 | 1.816 | 1.72 | 1.817 | 1.63 ± 0.3a,b |
| Menhinick | Ice cream factory | 0.6548 | 0.6988 | 0.6359 | 0.6858 | 0.67 ± 0.03a |
|  | Cheese factory | 0.4355 | 0.7195 | 0.5843 | 0.7451 | 0.62 ± 0.14a |
|  | Biscuit factory | 0.3785 | 0.82 | 0.4829 | 0.7908 | 0.62 ± 0.22a |
|  | Chocolate factory | 0.3409 | 0.5357 | 0.2741 | 0.583 | 0.43 ± 0.15b,c |
|  | Meat factory | 0.2752 | 0.4851 | 0.3265 | 0.3758 | 0.37 ± 0.09c,d |
|  | Chips and corn factory | 0.08079 | 0.3771 | 0.2244 | 0.322 | 0.25 ± 0.13d |
|  | Onion factory | 0.1948 | 0.5853 | 0.2115 | 0.2435 | 0.31 ± 0.19c,d |
|  | Car’s factory | 0.2967 | 0.5518 | 0.5898 | 0.8721 | 0.58 ± 0.24a,b |
| Margalef | Ice cream factory | 2.778 | 4.053 | 3.468 | 3.739 | 3.51 ± 0.54a |
|  | Cheese factory | 2.081 | 4.084 | 3.095 | 3.924 | 3.3 ± 0.92a,b |
|  | Biscuit factory | 1.374 | 3.824 | 2.037 | 3.37 | 2.65 ± 1.14b,c |
|  | Chocolate factory | 1.222 | 2.522 | 1.146 | 2.583 | 1.87 ± 0.79d,e |
|  | Meat factory | 0.927 | 1.933 | 1.206 | 1.371 | 1.36 ± 0.42e |
|  | Chips and corn factory | 0.5804 | 3.578 | 2.027 | 2.935 | 2.28 ± 1.3c,d |
|  | Onion factory | 0.8376 | 3.779 | 0.9634 | 1.312 | 1.72 ± 1.39d,e |
|  | Car’s factory | 0.7081 | 1.786 | 1.825 | 2.694 | 1.75 ± 0.81d,e |
| Individuals | Ice cream factory | 933 | 2097 | 1803 | 1788 | 1655.25 ± 502.08b |
|  | Cheese factory | 1350 | 1978 | 1687 | 1621 | 1659 ± 257.86b |
|  | Biscuit factory | 698 | 1166 | 965 | 921 | 937.5 ± 192.01c |
|  | Chocolate factory | 697 | 1258 | 1078 | 1062 | 1023.75 ± 235.26c |
|  | Meat factory | 647 | 833 | 760 | 708 | 737 ± 78.92c,d |
|  | Chips and corn factory | 5515 | 7657 | 7172 | 7031 | 6843.75 ± 925.52a |
|  | Onion factory | 1291 | 2805 | 1431 | 2040 | 1891.75 ± 690.22b |
|  | Car’s factory | 284 | 473 | 414 | 380 | 387.75 ± 79.12d |
| Fisher_alpha | Ice cream factory | 3.595 | 5.358 | 4.504 | 4.916 | 4.59 ± 0.75a |
|  | Cheese factory | 2.55 | 5.422 | 3.963 | 5.226 | 4.29 ± 1.33a,b |
|  | Biscuit factory | 1.654 | 5.162 | 2.521 | 4.507 | 3.46 ± 1.65b,c |
|  | Chocolate factory | 1.458 | 3.175 | 1.346 | 3.287 | 2.32 ± 1.06d,e |
|  | Meat factory | 1.097 | 2.391 | 1.434 | 1.649 | 1.64 ± 0.55e |
|  | Chips and corn factory | 0.6649 | 4.426 | 2.37 | 3.558 | 2.75 ± 1.63c,d |
|  | Onion factory | 0.9734 | 4.877 | 1.118 | 1.528 | 2.12 ± 1.85d,e |
|  | Car’s factory | 0.862 | 2.24 | 2.31 | 3.652 | 2.27 ± 1.14d,e |
| Evenness_e^H/S | Ice cream factory | 0.2281 | 0.2301 | 0.2159 | 0.2705 | 0.24 ± 0.02b,c |
|  | Cheese factory | 0.169 | 0.1827 | 0.181 | 0.1467 | 0.17 ± 0.02c |
|  | Biscuit factory | 0.236 | 0.2561 | 0.3224 | 0.222 | 0.26 ± 0.04b,c |
|  | Chocolate factory | 0.2767 | 0.2798 | 0.4631 | 0.2605 | 0.32 ± 0.1b |
|  | Meat factory | 0.3493 | 0.2495 | 0.3087 | 0.2632 | 0.29 ± 0.05b |
|  | Chips and corn factory | 0.3575 | 0.09161 | 0.1358 | 0.09745 | 0.17 ± 0.13c |
|  | Onion factory | 0.3936 | 0.1815 | 0.3791 | 0.2628 | 0.3 ± 0.1b |
|  | Car’s factory | 0.6521 | 0.5123 | 0.4655 | 0.362 | 0.5 ± 0.12a |
| Equitability_J | Ice cream factory | 0.5066 | 0.5761 | 0.5349 | 0.6118 | 0.56 ± 0.05b |
|  | Cheese factory | 0.3587 | 0.5095 | 0.4622 | 0.4357 | 0.44 ± 0.06d |
|  | Biscuit factory | 0.3728 | 0.5912 | 0.582 | 0.5264 | 0.52 ± 0.1b,c,d |
|  | Chocolate factory | 0.4153 | 0.5674 | 0.6496 | 0.5431 | 0.54 ± 0.1b,c |
|  | Meat factory | 0.4594 | 0.4739 | 0.4651 | 0.4203 | 0.45 ± 0.02c,d |
|  | Chips and corn factory | 0.426 | 0.3164 | 0.3218 | 0.2935 | 0.34 ± 0.06e |
|  | Onion factory | 0.5208 | 0.5031 | 0.5335 | 0.4427 | 0.5 ± 0.04b,c,d |
|  | Car’s factory | 0.7344 | 0.7309 | 0.6923 | 0.6413 | 0.7 ± 0.04a |
| Dominance_D | Ice cream factory | 0.3312 | 0.2458 | 0.2953 | 0.205 | 0.27 ± 0.06c |
|  | Cheese factory | 0.4449 | 0.2904 | 0.3446 | 0.3556 | 0.36 ± 0.06b |
|  | Biscuit factory | 0.493 | 0.2415 | 0.3111 | 0.3151 | 0.34 ± 0.11b |
|  | Chocolate factory | 0.5031 | 0.2624 | 0.2974 | 0.2833 | 0.34 ± 0.11b |
|  | Meat factory | 0.4829 | 0.4078 | 0.4557 | 0.474 | 0.46 ± 0.03a |
|  | Chips and corn factory | 0.5283 | 0.429 | 0.4551 | 0.4579 | 0.47 ± 0.04a |
|  | Onion factory | 0.4187 | 0.2557 | 0.4432 | 0.3916 | 0.38 ± 0.08b |
|  | Car’s factory | 0.3608 | 0.2328 | 0.2494 | 0.2458 | 0.27 ± 0.06c |
| Brillouin | Ice cream factory | 1.47 | 1.958 | 1.725 | 2.018 | 1.79 ± 0.25a |
|  | Cheese factory | 0.9703 | 1.727 | 1.436 | 1.44 | 1.39 ± 0.31b,c |
|  | Biscuit factory | 0.831 | 1.912 | 1.536 | 1.613 | 1.47 ± 0.46b |
|  | Chocolate factory | 0.8849 | 1.635 | 1.406 | 1.559 | 1.37 ± 0.34b,c |
|  | Meat factory | 0.8706 | 1.213 | 0.9957 | 0.9388 | 1 ± 0.15d,e |
|  | Chips and corn factory | 0.7604 | 1.096 | 0.9413 | 0.9586 | 0.94 ± 0.14e |
|  | Onion factory | 1.002 | 1.701 | 1.095 | 1.05 | 1.21 ± 0.33c,d |
|  | Car’s factory | 1.142 | 1.753 | 1.653 | 1.721 | 1.57 ± 0.29b |
| Berger-Parker | Ice cream factory | 0.4523 | 0.3915 | 0.4287 | 0.3154 | 0.4 ± 0.06d |
|  | Cheese factory | 0.4748 | 0.3948 | 0.4161 | 0.4226 | 0.43 ± 0.03c,d |
|  | Biscuit factory | 0.5974 | 0.3902 | 0.4549 | 0.4658 | 0.48 ± 0.09c |
|  | Chocolate factory | 0.6428 | 0.4038 | 0.4397 | 0.4397 | 0.48 ± 0.11c |
|  | Meat factory | 0.5981 | 0.5618 | 0.5908 | 0.6045 | 0.59 ± 0.02a |
|  | Chips and corn factory | 0.6566 | 0.5062 | 0.5308 | 0.5453 | 0.56 ± 0.07a,b |
|  | Onion factory | 0.5755 | 0.3476 | 0.6296 | 0.4417 | 0.5 ± 0.13b,c |
|  | Car’s factory | 0.4507 | 0.3594 | 0.3647 | 0.3658 | 0.39 ± 0.04d |
| ACE | Ice cream factory | 23.41 | 43.34 | 28.35 | 29 | 31.03 ± 8.58a |
|  | Cheese factory | 18.31 | 37.16 | 27.78 | 32.04 | 28.82 ± 7.99a |
|  | Biscuit factory | 10.85 | 32.07 | 15 | 25.2 | 20.78 ± 9.64b,c |
|  | Chocolate factory | 9 | 20.96 | 9.516 | 20.56 | 15.01 ± 6.65c,d |
|  | Meat factory | 7 | 19.73 | 10.6 | 14.62 | 12.99 ± 5.47d |
|  | Chips and corn factory | 6 | 34.87 | 19.36 | 27.98 | 22.05 ± 12.44b |
|  | Onion factory | 7.571 | 32.3 | 8.516 | 19.1 | 16.87 ± 11.54b,c,d |
|  | Car’s factory | 5 | 12 | 13.57 | 17.62 | 12.05 ± 5.26d |

*Means that do not share a letter are significantly different.*
